# Between uncertainty and destiny: the patient journey in axial spondyloarthritis care from the perspectives of patients and their relatives

**DOI:** 10.1186/s41927-025-00465-3

**Published:** 2025-02-12

**Authors:** Susann May, Greta Nordmann, Franziska Gabb, Katharina Boy, Magali Wagner, Niklas Ohm, Hanna Labinsky, Johannes Knitza, Sebastian Kuhn, Martin Heinze, Martin Welcker, Felix Muehlensiepen

**Affiliations:** 1 Center for Health Services Research, Faculty for Health Sciences, Brandenburg Medical School, Rüdersdorf, Germany; 2 Rheumatology/Clinical Immunology, Department of Internal Medicine II, University of Würzburg, Würzburg, Germany; 3 Institute for Digital Health, University Hospital of Giessen and Marburg, Philipps-University Marburg, Marburg, Germany; 4 AGEIS, Université Grenoble Alpes, Grenoble, France; 5 Department of Psychiatry and Psychotherapy, Brandenburg Medical School, Immanuel Klinik Rüdersdorf, 15562 Rüdersdorf, Germany; 6 MVZ for Rheumatology Dr. Martin Welcker GmbH, Planegg, Germany

**Keywords:** Rheumatology, Axial spondyloarthritis, Qualitative research, Patient journey, Family care giver

## Abstract

Axial spondyloarthritis (axSpA) is an inflammatory rheumatic disease primarily affecting the sacroiliac joints and spine, leading to chronic pain, fatigue, and reduced mobility. The diagnostic delay for axSpA is often long, causing significant physical, psychological, and social burdens for patients and their relatives. This study aims to explore the patient journey of individuals with axSpA and their relatives within the German healthcare system, identifying key challenges and unmet needs from symptom onset to diagnosis and treatment. A qualitative approach was employed, involving structured interviews with axSpA patients and their relatives. Participants were selected through purposive sampling to ensure diverse representation. Data collection involved individual telephone interviews, which were transcribed and analyzed using Kuckartz’s structured qualitative content analysis framework. The patient journey was characterized by four distinct phases: Time before diagnosis, Diagnosis, After the diagnosis, and Current treatment. Participants reported significant psychological and emotional burdens, with many experiences attributed to chance encounters with knowledgeable healthcare professionals. Key issues included a lack of awareness among healthcare professionals, diagnostic delays, and inadequate psychological support. The perspectives of patients and their relatives highlighted the significant psychological burden they both experience throughout the journey. This underscores the need for services that cater not only to patients but also to their relatives. The study highlights critical gaps in the current healthcare system regarding the diagnosis and care of axSpA patients. To improve care, systematic efforts are needed to enhance awareness, reduce diagnostic delays, integrate psychological support, and provide comprehensive information throughout the patient journey for both, patients and relatives. Effective care should not rely on chance; systematic improvements are necessary to ensure consistent, high-quality care.

**Supplementary Information:**

The online version contains supplementary material available at 10.1186/s41927-025-00465-3.

## Introduction

Axial spondyloarthritis (axSpA) is an inflammatory rheumatic disease (IRD), primarily affecting the sacroiliac joints and spine, often with additional articular and extra-articular involvement [1]. This condition is characterized by chronic low back pain, fatigue, and reduced mobility [1], significantly impacting patients’ physical, psychological, and social well-being, including their work roles and personal relationships [2]. The Assessment of SpondyloArthritis international Society (ASAS) characterize axSpA as a potentially severe disease with diverse manifestations, usually requiring multidisciplinary management coordinated by the rheumatologist [3].

In addition to the complexity of the clinical picture, however, the declining number of employees in rheumatology and the increasing demand for consultations represent a major challenge for early detection [4, 5]. The recent IMAS (International Map of Axial Spondyloarthritis) survey reported a mean diagnostic delay was 7.4 ± 9.0 years (median: 4.0) for axSpA [6]. For German patients with suspected rheumatic diseases before special care consultation, we identified axSpA as the IRD with the longest diagnostic-delay with 2.9 ± 5.9 years (median: 0,96) in a recent multi-centre based study [7]. This delay is influenced by disease-related factors, referring physicians and, patient-related factors [8–12]. The period between the onset of symptoms and diagnosis is described as meandering and frustrating [8], significantly impacting the well-being and mental state of both patients [13] and their relatives.

While several qualitative studies have examined diagnostic delays and other aspects of the patient journey for axSpA from the perspectives of patients and healthcare professionals (HCPs) [7–13], the views of relatives—a crucial part of the patient’s support system—remain unexplored. This is a significant research gap, as evidence from other medical fields indicates that relatives often bear a considerable psychological burden during the patient journey [14]. They also have distinct information needs [15] and require specific forms of support [16], which can directly influence the patient’s overall experience and outcomes.

Thus, the primary objective of this project is to explore the perspectives of patients and their relatives on the patient journey with axSpA in German health care. By mapping the trajectory from symptom onset to diagnosis and treatment, this study aims to identify key challenges and unmet needs during the diagnostic process and beyond. These insights will inform HCPs, policymakers and patient organisations about critical areas requiring attention, ultimately improving the quality of life for those affected by axSpA, both patients and relatives.

## Methods

To gain data on the perspectives of patients and their relatives on the patient journey with axSpA in German health care, a qualitative approach was selected for data collection and analysis [17]. We chose individual qualitative interviews to allow participants to discuss sensitive personal and family-related experiences in a confidential setting. The aim was to gain a comprehensive understanding of the patient journey and associated challenges and to systematize them accordingly.

### Participants and sampling

Participants were selected using purposive sampling [18]. A purposeful selection, considering age, civil status, educational level, employment, and duration of the disease was made in order to attain variation among the participants [19]. The inclusion criteria were: axSpA patient or relative of an axSpA patient, age ≥ 18, native German speaker and willingness to participate in the study. Patients were recruited via an article in the member magazine of the patient organisation Deutsche Vereinigung Morbus Bechterew e.V. and via members of their youth group Netzwerk Junge Bechterewler. Participants were also asked to arrange contact with their relatives (with or without axSpA diagnosis). Participants were selected over a period of three months, consistent with available resources. We chose to conduct 20 interviews as we expected this number to reach theoretical saturation. Saturation was defined as code saturation, indicating no additional issues identified, and meaning saturation, indicating no further dimensions, nuances, or insights could be found [20]. The participants received no incentives for their participation.

### Data collection

Data was collected through individual telephone interviews (FG). The interviews took place between April and August 2023. Each interview was audio-recorded and transcribed verbatim. A preliminary semi-structured interview guide was drafted by a multiprofessional team (SM, FM, FG) specifically for this study. The semi-structured interview guide focused on exploring the initial manifestation of the illness, the patient’s first contact with the healthcare system, their experience receiving an initial diagnosis, the subsequent healthcare process they navigated, and their specific needs for information and support throughout their journey. In addition, socio-demographic data were collected.

The interview guide was tested with two patients to ensure clarity and relevance of the questions, allowing to refine the instrument based on preliminary feedback. This process helped in establishing a reliable and effective tool for eliciting in-depth insights from participants in the main study phase. After piloting there was no need to revise the final interview guide. For detailed information on the interview guide, please refer to the Supplementary Material 1. Prior to providing verbal informed consent, participants were afforded the opportunity to pose any queries they had. Following the interviews, participants were expressed gratitude for their time and given the chance for further inquiries. Subsequently, the interviews were transcribed verbatim and anonymized. Participants did not receive the transcripts for feedback or correction. These transcripts were then imported into MAXQDA Analytics Pro 2022, Release 22.1.0 from Verbi GmbH (Berlin, Germany) to record field notes and support data analysis.

### Data analysis

The interview data underwent a detailed analysis conducted by members of the research team (SM, FM, GN) utilizing Kuckartz’s structured qualitative content analysis as theoretical framework [17]. Interviews from patients and relatives were analyzed together to identify overarching themes.

The analysis commenced with meticulous examinations of ten interview transcripts, which led to the initial coding process. Segments of the interviews were classified using both a deductive and an inductive approach. The iterative process of inductive reasoning allowed for the formation of primary and secondary categories from these codes. The research team engaged in thorough discussions until a consensus on the categories was achieved, ensuring a cohesive interpretation across the team. Certain categories were also pre-formulated in alignment with the aims of the study and subsequently incorporated into the existing coding structure. The initial coding and interpretation process of the ten interviews resulted 7 primary categories and 40 secondary categories.

After completion of the data collection, two researchers (SM, FM) independently applied the established category system to analyze the entire dataset, ensuring transparency and reproducibility. The results of the analysis were presented to and confirmed by participants in a videoconference. For the dissemination of the research findings, pertinent sections of the transcripts were chosen to serve as illustrative quotes. These selected quotations were then translated into English to be included in the manuscript, adhering to the Consolidated Criteria for Reporting Qualitative Research (COREQ) please refer to the Supplementary Material 2 standards [21].

## Results

### Participants’ characteristics

Twenty-three interviews with 16 patients and 7 relatives were conducted and analyzed until theoretical saturation was reached. Mean age of interviewed participants was 49 (range: 27–75) years, see Table 1. 12/23 (52%) of the participants were female. Participants reported diverse occupational and educational backgrounds. All patients had a suspected axSpA diagnosis. The interviews lasted between 19 and 52 min (mean 34,9 min).

**Table 1:** Participant characteristics

Participant ID	Patient or relative	Age	Gender	Level of education	Occupation
1	Relative	51	Female	High school diploma	Unemployed
2	Relative	47	Female	University degree	Public service employee
3	Relative	27	Male	Vocational training	Pensioner
4	Relative	28	Female	High school diploma	Administrative assistant
5	Patient	52	Female	Secondary school diploma	Speech and language therapist
6	Patient	51	Male	University degree	Pensioner
7	Patient	63	Female	Vocational training	Pensioner
8	Relative	56	Female	Secondary school diploma	Speech and language therapist
9	Patient	46	Female	Vocational training	Practice management medical care center
10	Patient	53	Male	Secondary school diploma	Administrative assistant
11	Patient	27	Male	Vocational training	Self-employed
12	Patient	35	Female	University degree	Employee in PR and marketing
13	Patient	42	Female	Vocational training	Accounting Manager
14	Patient	66	Male	University degree	Psychologist
15	Patient	35	Male	University degree	Emergency paramedic
16	Patient	55	Male	University degree	Architect
17	Patient	43	Male	University degree	Civil servant
18	Patient	75	Male	Vocational training	Pensioner
19	Patient	53	Female	Secondary school diploma	Office administrator
20	Patient	46	Male	University degree	Employee
21	Patient	62	Female	Secondary school diploma	Pensioner
22	Relative	65	Male	University degree	Pensioner
23	Relative	55	Female	Vocational training	Pensioner

### Patient journey

**Fig. 1:**
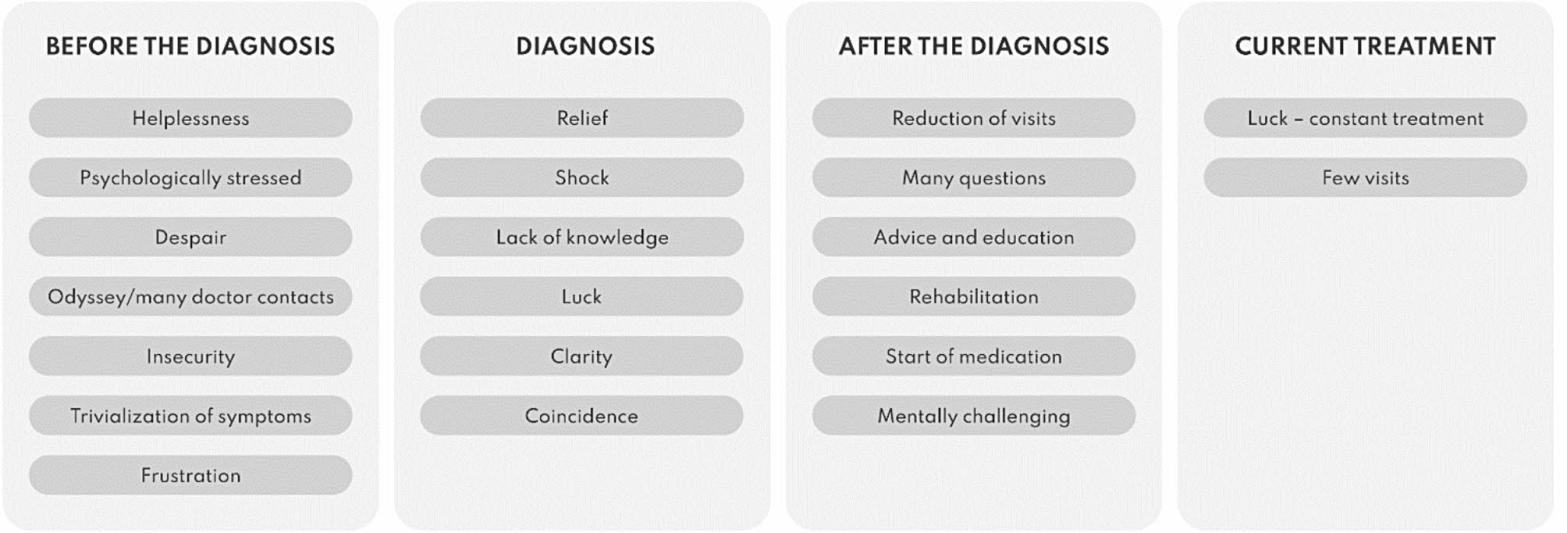
Four segments of the patient journey in axSpA (coding tree)

The qualitative data reveals that the patient journey in axSpA can be categorized into four segments (Fig. 1).

### Time before diagnosis

The time before the diagnosis of axSpA is often characterized by profound **perplexity and helplessness** on the part of the patients and also their relatives. Unspecific symptoms like chronic back pain and stiffness lead to confusion and **insecurity**. Patients, relatives and HCPs are equally confronted with the challenge of identifying the cause of the symptoms. The lack of clarity during this phase increases the feeling of helplessness.

Of course it was a big frustration, I think for both, my wife and me. For her, of course, I think it’s always much broader than for me, because let me say, of course I’m the relative now, but I don’t have the pain myself. And I think there’s just this perplexity, also helplessness, at the moment when you think that this can’t be happening. She was in her mid-to-early 20s at the time. And to be so limp already, let me say, with your own body, is of course really, really difficult. And that’s why there was definitely a strong sense of helplessness. Because the doctors couldn’t say, hey, okay, that’s it and now let’s just try to treat something. There was still no way or goal to achieve. (3_Relative, Pos. 13)

The diagnostic journey is commonly described as an “odyssey”. Patients are repeatedly referred from one physician to another and subjected to numerous examinations without obtaining definitive answers. They endure a multitude of consultations across various specialties. This phase is experienced as both physically and emotionally taxing, profoundly impacting the quality of life of patients and their families.

I also started a bit of an odyssey again before the diagnosis, but in between I didn’t actually go to the doctor because of it and I know that it was a bit of a mixture of frustration, a high threshold to go to the doctor again, because they couldn’t find anything and dismissed it, and a very big repression like: Okay, they say that can’t be right, and I don’t see anyone around me who behaves, in brackets, like me, so it’s probably nothing and I just have to grit my teeth and that’s just how life is now. (16_Patient, Pos. 27)

The entire time before the diagnosis is characterized by **psychological stress** for both, patients and relatives. The uncertainty about the cause and the future leave emotional traces. Psychological distress can take various forms, from fear and insecurity to frustration and depression.

That was of course extremely stressful. (11_Patient, Pos. 63)

At some point you believe yourself that you’re imagining it, right? (18_Patient, Pos. 12)

Another challenging aspect before making a diagnosis is that doctors tend to trivialize the symptoms from the patient’s and relatives’ perspective. Doctors assessed the symptoms as temporary or less significant.

I still remember it well, that at some point we had an appointment at the clinic in [city], an outpatient appointment, where you look at the whole picture, but even at this appointment there was somehow the question, well,[…] she would have to relax a little more. (2_Relative, Pos. 16)

And then my hand was patted and they said, that I was a very extroverted guy, I should calm down, I should do Yoga and then things will be fine again. (14_Patient, Pos. 21)

The entire time before the diagnosis is experienced as **frustrating** by both the patients and their relatives.

And she’s basically experiencing similar stories as many, many others. No diagnosis for years, not be taken seriously. That’s actually pretty frustrating. (2_Relative, Pos. 66)

### Diagnosis

The certainty about the cause of the long-lasting symptoms brings **relief** to the patients and their relatives. After a long phase of uncertainty, the diagnosis means the end of uncertainty and powerlessness.

And with the diagnosis I was quite relieved at first, because then … well, I now have it black on white, there’s something wrong, there’s something. In fact, it’s not me who’s wrong, but there’s a reason. And that left me quite relieved at first. (16_Patient, Pos. 45)

In addition, those affected reported the aspect of **clarity that they did not simulate**.

Because once you knew, so when the shock had been digested and the child had a name and you didn’t think that you were somehow like a hypochondriac and were imagining something, but you finally knew what it was then of course you could live according to it. (18_Patient, Pos. 77)

Some patients and their relatives felt **shocked** by the diagnosis. The reality of being confronted with a chronic illness led to a state of emotional turmoil for those affected. In particular, realizing that future life plans and activities may be affected by the disease.

When the doctor told me this, my first thought was, that I would never be able to dance with my daughters-in-law at my sons’ weddings because at that time I worked for a health insurance company and I was familiar with a lot of symptoms or illnesses. And as I said, my stance at the time was, yes, these are the people whose spine is stiff, who walk around all day as if they were looking for water on the ground. That was my first thought about it. (15_Patient, Pos. 25)

This feeling is not only experienced by the patients, but also by their relatives.

Exactly, above all it’s chronic, it’s not curable. That means, it won’t be for a few weeks now, a few months and he is healthy again. No, the disease is there, it can even get worse. The best you can do is try to stop it a little, but it will never go away. And this fact alone, this thing is here now and that it will never get better. It can only get worse, but it will never ever get better. Also psychologically, I would say, it’s incredibly difficult. (1_Relative, Pos. 7)

The immediate reaction to the diagnosis often included **lack of knowledge** about the disease itself and its treatment options.

When I got the diagnosis, I couldn’t really do anything with it and I didn’t know at all, what was expecting me and then I kind of had a mental breakdown. (18_Patient, Pos. 43)

Participants described the diagnosis as **surprising or coincidental**, often occurring due to serendipitous encounters with HCP. These included being referred to specialists by chance, having routine tests reveal unexpected results, or meeting doctors with prior knowledge of axSpA. For example, one patient reported that a rheumatologist happened to notice signs during a routine consultation, leading to further diagnostic steps.

And that [the diagnosis] was basically a coincidence, that’s what I would call it. (3_Relative, Pos. 29)

The period of diagnosis differed in two ways. Either the diagnosis was made quite quickly within a few months, or a long time passed before the diagnosis was made. Patients sometimes had to wait for years to be diagnosed. Patients who were diagnosed quickly spoke of how **lucky** they were that the diagnosis was made quickly.

That was really a lot of luck, because others have issues with this for years. (4_Relative, Pos. 15)

The aspect of the luck was also thematized, when speaking about getting an appointment at a specialist for diagnostics.

He [the rheumatologist] took me in immediately, so I was very lucky, I must say. (10_Patient, Pos. 5)

In addition, the aspect of luck was addressed when it came to finding a doctor who made a suspected diagnosis and referred to experts.

People don’t always take you so seriously, but I remained very persistent and then I was just lucky that I ended up with an orthopedic surgeon who said, there’s something wrong here, it’s not normal, we do the HLA-B27 test. Then he basically referred me to a rheumatologist. (18_Patient, Pos. 27)

### After the diagnosis

Before the diagnosis is made, many specialists are often involved in the care process. Once the diagnosis is made, **contact with doctors is reduced** to the bare essentials.

With the diagnosis the doctor-racing also stopped. (14_Patient, Pos. 117).

After the diagnosis was made, all participants went through a phase in which a **large number of questions** arose. It was often emphasized how important having good medical care that focused on advice and information was.

So let me say, an X-ray or I don’t know what kind of wind, you can see a lot, but you don’t understand anything. Even if you read through something, there is a lot of foreign language that you, as a craftsman, tend to have no idea about. And you need a very, very good doctor or very, very good advice in order to get an overview. (3_Relative, Pos. 25)

In most cases, patients took rehabilitative measures. Here they reported that they were provided with extensive information about the disease and were comprehensively educated. In addition, **medication was started** in this phase.

During this phase, most patients began to come to terms with their illness. Relatives described the period following the diagnosis as particularly **psychologically challenging**, especially for themselves.

I was almost in the same helpless position, just without this pain, and that’s, I think … I think it was a very difficult time for my wife physically and mentally, and for me it was just mentally. Because it’s also the case that you plan to do certain things, plan them and then she just says, it won’t work, it won’t work. Then of course you already have a little … you need to be understanding and say, well, then it just won’t work. (3_Relative, Pos. 47)

### Current treatment

Those affected and their relatives reported that axSpA care is typically limited to a few consultations with a rheumatologist. Good rheumatology care is described as a matter of luck.

And so far my wife has been very, very lucky because the rheumatologist is very, very good and he was able to explain everything to her in more detail and really explain things calmly and understandably in a way that made sense. (3_Relative, Pos. 35)

It is repeatedly reported that it is sometimes difficult to find a rheumatologist who can provide care. For example, relatives reported disruptions in benefits when doctors retired.

It was difficult in between when the doctor retired from the clinic and the clinic then had a new doctor … so she was very worried that she would somehow go backwards again or that the care (…). (2_Relative, Pos. 52)

### Support needs of patients with axSpA and their relatives

**Fig. 2:**
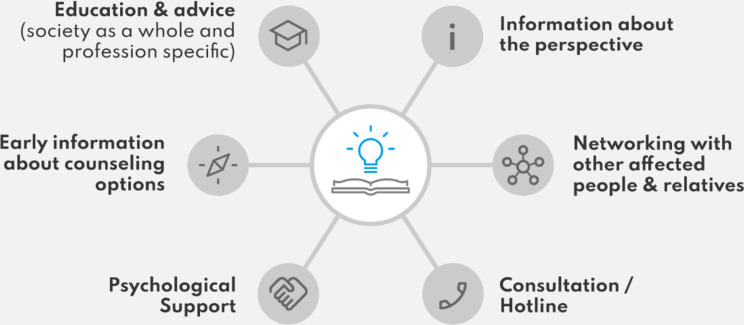
Support needs of patients with axSpA and their relatives (coding tree)

The complex needs of patients with axSpA and their relatives require a multidimensional approach that goes beyond purely medical treatment. Figure 2 illustrates the wishes for support services from the perspective of patients and their relatives.

From the perspectives of patients and their relatives, there is a deficit in public knowledge and awareness about axSpA. In order to address this, comprehensive educational measures and awareness campaigns are required for society as a whole that enable the dissemination of information about the disease, demystification of prejudices and the promotion of a well-founded understanding.

Well, as I said, I would like the education to be there because the education would also mean more acceptance from those around us. All you hear is: “What’s the matter? You’re moving, you’re going for a run, you’re not sick, now don’t act like that.” That has nothing to do with it. Just because you can’t see an illness, because I don’t have a cast on my leg or something like that, that still means that I’m sick. This is also the case with mentally ill people and I find that a bit sad because hardly anyone says, yes, my husband has it … “What?! What is that?” So no one can imagine anything about it. And no one understands it because everyone only sees the one who is running. (1_Relative, Pos. 83)

Lacking awareness, education and sensitization towards axSpA has also been discussed with regard to HCP:

Looking at all the years, I would have simply wished that people would have paid more attention to the signs and that people would have taken my son more seriously and looked more closely at what was actually going on. I would have liked that, then he would have had more certainty for many years and not had the feeling of ‘no one believes me anyway. (13_Relative, Pos. 99)

From the respondents’ perspective, early access to comprehensive information about available advisory services is crucial, particularly in the initial stages of their journey before receiving a formal diagnosis or being referred for rehabilitation.

The importance of **psychological support** as an integral part of the care of axSpA-patients, but also of their relatives, is mentioned by the participants as an important support offer.

So maybe what I would wish for, is that there would be some kind of seminar or something where you can learn how to deal with mental health problems. Because if you have severe pain over and over again, then of course it affects your mental health and how you as a partner can deal with it, how I can help him in some way, for example. It would be nice if there were somehow offers to learn something like that. (4_Relative, Pos. 47)

Patients and relatives address the desire for transparent information regarding the prognostic **perspectives of the disease**. From their point of view, clear communication about long-term forecasts, possible developments and options for the future are essential in order to minimize uncertainties.

Maybe more information about what the status is and what else should we expect? What else should we expect? (14_Patient, Pos. 97).

From the participants’ perspective, **the opportunity to network with those affected and their relatives** through patient organizations, self-help groups or digital platforms such as social media (Instagram or similar) creates a supportive community.

I also looked a lot on Instagram in the preliminary phase. This is actually not the best source, but you can also see a lot of people there who have this disease. (17_Patient, Pos. 71)

Additionally, participants expressed a desire for face-to-face seminars that provide detailed information in a group setting, offering opportunities to exchange experiences with other patients, relatives, and HCPs.

And of course also inform yourself, because you get completely different perspectives from experienced people, from people who are affected by it, and then everyone gives their experiences and their tips. The same thing doesn’t help everyone, but then you might just get a little something out of it where you say, yes, that’s right, of course I could try that too, and that’s possible, oh look, I didn’t know that. Of course, exchange ideas with each other. This is really important, but you could have started this much earlier if you had known what you have. (1_Relative, Pos. 93)

The relatives should be addressed directly, so that they would have a sense of recognition.

Yes, and if it perhaps explicitly says for relatives. Otherwise I would now have the feeling that if I was researching something, ah yes, okay, it’s just about the, in brackets, just in quotation marks, the sick person. I feel like I’m crossing a line. But if it says: “Support for relatives of…” I think I saw that recently in Munich at a tram stop, relatives of people with mental problems. Then you would think, ah yes, okay, that’s me, that’s exactly what I want. Maybe a little more targeted. (16_Patient, Pos. 65)

The respondents believe that implementing a dedicated **consultation hour or hotline** for axSpA patients and their relatives would be beneficial in order to be able to ask care-related questions at short notice without having to wait a long time for an appointment with a doctor.

I mean, you’ve already asked the question earlier, what would I want or something like that? If I look at it under this aspect, of course, I would like there to be some kind of consultation hour that doesn’t necessarily just deal with medication or in a medical context (…) Yes, or a hotline where you can call and where you can get expert knowledge or at least information, where you can get it. I mean, information is very diverse and it’s not just reduced to one illness. There are always questions, maybe even questions from relatives, but I don’t even know where to go. Well, except that I’m at the doctor’s appointment and can ask the doctor. (2_Relative, Pos. 104 & 106)

**Fig. 3:**
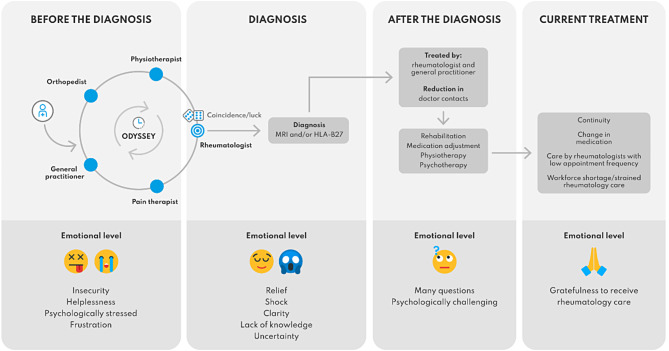
Map of the patient journey in axSpA

Based on the results of the qualitative content analysis, we were able to create a comprehensive map of the patient journey for patients and their relatives (Fig. 3), which takes into account the various phases and needs of both patients and their families.

## Discussion

This qualitative study aimed to explore the perspectives of patients and their relatives on the patient journey in axSpA within the German healthcare system. By mapping the trajectory from symptom onset to diagnosis and treatment, the study sought to identify key challenges and unmet needs during the diagnostic process and beyond.

### Principal findings

The journey from symptom onset to diagnosis and treatment, as described by patients and their relatives, was characterized by its length and a pervasive sense of confusion and helplessness. This often led to both physical and emotional exhaustion. Participants emphasized that information needs vary significantly throughout the journey. In the early stages, access to clear and accurate information about symptoms and potential diagnoses is vital. Following diagnosis, comprehensive guidance on long-term disease management becomes essential.

The narratives revealed a significant psychological burden experienced by both patients and their relatives, highlighting the importance of support services that address the needs of both groups. Notably, positive outcomes were frequently attributed to chance encounters, such as meeting knowledgeable HCP or accessing effective rheumatology care. This dependence on chance encounters underscores the urgent need for systematic improvements to ensure consistent, high-quality care that does not depend on luck.

Participants’ accounts also reflected systemic issues, such as inadequate access to specialized rheumatology care and gaps in physician training. For instance, diagnostic delays were often linked to a lack of awareness among general practitioners regarding axSpA symptoms. Beyond individual experiences, these findings highlight broader systemic challenges that extend to chronic disease management. Addressing these challenges requires targeted interventions, such as improving referral pathways, implementing interdisciplinary collaboration, and leveraging digital health solutions.

### Comparison with earlier results

Our results are in line with previous results on the patient journey in axSpA. In our interviews, participants described it as a matter of chance or luck if they HCP who could explain their symptoms and diagnose axSpA. These findings highlight the results of previous studies on the lack of awareness [10–13] and the need for initiatives to increase axSpA awareness among HCP [3, 22].

Our findings confirm the results of a British interview study by Martindale et al. [9], which divided the patient journey in axSpA into four phases in their thematic analysis: ‘What’s going on?’ described the process of attempting to comprehend a fluctuating and severe back pain experience. ‘Fighting for a diagnosis’ provided insights into the struggle to be taken seriously and the feeling of being dismissed by HCPs. ‘Being adrift’ explored the negative psychological effects linked to the search for a diagnosis. ‘The start of a journey’ described the relief of receiving a diagnosis, contrasted with the emotions tied to a long-term degenerative condition. In our qualitative data, we were also able to identify four distinct phases of the patient journey with very similar patient reported experiences, which overlap with the findings of Martindale et al.

Similarly, Dube et al.’s qualitative study among US axSpA patients [8] found that the prolonged time until diagnosis was associated with significant frustration and mental distress. Our findings reveal that relatives of patients with axSpA also endure considerable burden. This experience is comparable to other medical fields, such as oncology, and others [14–16], where specific support programs for relatives are in place [23]. These programs could serve as a valuable model for developing similar support structures for axSpA care.

### Strengths and weaknesses of the study

The inclusion of relatives’ perspectives represents a major strength of this study, addressing a critical gap in the literature. By exploring their experiences alongside those of patients, our findings provide a more comprehensive understanding of the axSpA care journey. Additionally, while there have been studies on diagnostic delay in axSpA in Germany, this is the first qualitative study on the patient journey in axSpA in Germany [24].

Yet, our study has several limitations. Most participants were recruited through a patient organization, selection bias. This may result in overrepresentation of highly engaged individuals with more severe symptoms or better access to resources. Future studies should aim to recruit participants through diverse channels, such as primary care settings, to capture a broader spectrum of experiences. Additionally, due to self-selection, it is possible that those who chose to participate were particularly motivated to share their experiences about the patient journey, which may not be representative of all axSpA patients and their relatives. Another limitation is that this study did not differentiate between non-radiographic and radiographic axSpA [24, 25]. Since the experiences and challenges of patients and their relatives may differ between these two subtypes, future research should address these distinctions to gain deeper insights. Additionally, we did not explicitly collect data on migration background, which is an important determinant that could influence the course of the patient journey. Furthermore, only native German speakers were included in this study. The experiences of individuals with a migration background and/or limited German proficiency may differ significantly compared to this sample, which predominantly represents a more engaged group. These are critical aspects for future studies, which should aim to include a more diverse sample and address these limitations to provide a broader and more inclusive understanding of the axSpA patient journey.

As researchers with backgrounds in rheumatology and health services research, our professional perspectives may have influenced the interpretation of data. While this multidisciplinary expertise enriched the analysis, we acknowledge the potential for unconscious biases. Efforts were made to mitigate this by engaging in team discussions and seeking participant feedback on preliminary findings.

### Implications for clinicians and policymakers

The findings from this study underscore the need for systematic improvements in the diagnosis and care of axSpA patients and their relatives: To enhance awareness of axSpA among HCP and the general public, it is essential to strengthen the work of patient organizations like the Deutsche Vereinigung Morbus Bechterew and the Deutsche Rheuma-Liga in Germany. Together with professional associations, these organizations play a crucial role in raising awareness and providing support for patients and their families. Implementing comprehensive educational campaigns aimed at HCP across various specialties and particularly for primary care can improve early recognition and diagnosis of axSpA. Efforts to reduce the diagnostic delay for axSpA should also include the development of new diagnostic pathways [26] and support for initiatives like the Horizon Europe funded SPIDeRR project [27] that focuses on developing and implementing early diagnostic tools and protocols.

Recognizing the psychological burden on both patients and their relatives, it is vital to ensure that psychological support services are integrated into the routine care of axSpA patients and their families. In this context, digital platforms can play a transformative role in providing accessible and scalable support. For example, the app Axia [28] supports home-based disease-specific exercise programs and aims to enhance patient knowledge, while the axSpA-Connect initiative by the Deutsche Vereinigung Morbus Bechterew [29] offers a networking platform for patients and relatives to share experiences and access tailored information. Despite their potential, these resources are not yet widely utilized. Barriers such as limited awareness, varying levels of digital literacy, and resistance to digital tools hinder their broader adoption.

To address these challenges, integrating digital platforms into standard care pathways is essential. Healthcare professionals should actively promote their use during consultations, and targeted campaigns should raise awareness among patients and families about these tools. Educational programs for HCPs on the effective use and recommendation of digital resources could further support their integration. Moreover, ensuring accessibility for underserved populations, such as individuals with limited digital skills or language barriers, is crucial for equitable implementation.

Developing comprehensive information databases and resources covering all aspects of axSpA management—available both digitally and in traditional formats—can further empower patients and their families. Systematic screenings, such as the axSpA-Knowledge Screening [30], could identify knowledge gaps and guide targeted education efforts for patients and HCPs.

Implementing these recommendations is not without challenges. Resistance among HCPs, driven by increased workload or insufficient training, may present obstacles. Resource limitations and institutional inertia could also hinder systemic change. To overcome these barriers, stakeholder engagement and pilot programs should be prioritized to demonstrate the feasibility and effectiveness of these measures. By addressing these challenges proactively, the healthcare system can move toward providing consistent, high-quality care for axSpA patients and their families.

## Conclusion

This study highlights the critical gaps in the current healthcare system regarding the diagnosis and care of axSpA patients. The journey from symptom onset to diagnosis is often prolonged and fraught with challenges, underscoring the need for systematic improvements. Enhancing awareness, early diagnosis, psychological support, and providing tailored information are essential steps to improve the care and quality of life for axSpA patients and their relatives.

## Electronic supplementary material

Below is the link to the electronic supplementary material.

Supplementary Material 1

Supplementary Material 2

## Data Availability

All data relevant to the study are included in the article or uploaded as supplementary information. For further questions regarding the reuse of data, please contact the corresponding author (felix.muehlensiepen@mhb-fontane.de).

## Abbreviations

ASAS: Assessment of SpondyloArthritis International Society
axSpA: Axial Spondyloarthritis
COREQ: Consolidated Criteria for Reporting Qualitative Research
HCP: Healthcare Professional
IMAS: International Map of Axial Spondyloarthritis
IRD: Inflammatory Rheumatic Disease
SPIDeRR: Stratification of patients using advanced integrative modelling of data routinely acquired for diagnosing rheumatic complaints
