# Supplementary material for: Between uncertainty and destiny: the patient journey in axial spondyloarthritis care from the perspectives of patients and their relatives
Source: BMC Rheumatol. 2025 Feb 12;9:14. doi: 10.1186/s41927-025-00465-3 (PMC11818133; doi:10.1186/s41927-025-00465-3)
Supplement: Supplementary file 1 — Supplementary Material 1 [file 41927_2025_465_MOESM1_ESM.pdf]

Guideline: Early Detection of SPondyloARThritides (SpA) based on multimodal Analysis of individual Disease Concepts and Approaches in those affected with Symptoms II – SPARTAKUS II

Key Question/Narrative Impulse	Check Aspects	Specific Questions	Maintenance and Control Questions
In our study, we are investigating what information and support relatives of people with Spondylarthritis need.<br><br>You suffer from Spondyloarthritis. Could you please tell me what it was like when the illness first appeared?	Manifestation<br><br>*Uncertainty*<br>*Ignorance*<br>*Overwhelm*	When was it?<br>How did the disease manifest itself?<br>Who supported you the most during that time (relatives)?<br>Who else supported you (relatives)?<br>How did your relatives learn about it?<br>How did your relatives deal with the illness?<br>What did you and your relatives do back then?<br>What did you and your family think back then?<br>How did you and your relatives feel back then?	Can you tell me more about that?<br><br>And then?<br>How was that for you?<br><br>How do you see it?<br>Can you please elaborate on this?<br><br>Could you please give an example?<br><br>What do you mean exactly?
When did you first come into contact with the healthcare system due to your illness?	Initial Contact with the Healthcare System	What kind of practice was that?<br>What kind of doctor was that?<br>What happened next?<br>Please describe that.	Can you tell us more about this?

Please describe.		How did you feel back then?<br>Did you have questions?	Can you tell us more about this?
When was diagnosis SpA first made?<br><br>How was that for you?	Initial Diagnosis	What kind of practice was that?<br>What kind of doctor was that?<br>What happened next?<br>Please describe that.<br>How did you feel back then?<br>Did you have questions?	And then? How was that for you?<br><br>How do you see it? Can you please elaborate on this?<br><br>Could you please give an example?<br><br>What do you mean exactly?
What happened next?<br><br>Please describe the furthercare process of SpA until today.	Healthcare Care Process		Can you tell us more about this?
If you now look back at the entire time, right up to the first appearance of the symptoms...	Need for Information and Support	… would you say that your relatives felt sufficiently supported?<br><br>Which offer of support were/would have been important for your relatives?<br><br>In what do you need support now? And what would help to make you feel better?<br><br>Which information did your relatives need and **when**? / would your relatives have needed and **when**?	Can you tell us more about this?

		Where did your relatives get information from?<br><br>Which wishes did you/do you and your relatives have?<br><br>How could the medical care process have been improved for you and your relatives?	

Age	Sex	When was the diagnosis made?
**Highest educational degree**	**Profession**	
